# Copy-Move Forgery Detection (CMFD) Using Deep Learning for Image and Video Forensics

**DOI:** 10.3390/jimaging7030059

**Published:** 2021-03-20

**Authors:** Yohanna Rodriguez-Ortega, Dora M. Ballesteros, Diego Renza

**Affiliations:** Faculty of Engineering, Universidad Militar Nueva Granada, Bogotá 110111, Colombia; est.yohanna.rodrig@unimilitar.edu.co (Y.R.-O.); diego.renza@unimilitar.edu.co (D.R.)

**Keywords:** copy-move forgery detection, computer vision, deep learning, fake image, transfer learning, VGG

## Abstract

With the exponential growth of high-quality fake images in social networks and media, it is necessary to develop recognition algorithms for this type of content. One of the most common types of image and video editing consists of duplicating areas of the image, known as the copy-move technique. Traditional image processing approaches manually look for patterns related to the duplicated content, limiting their use in mass data classification. In contrast, approaches based on deep learning have shown better performance and promising results, but they present generalization problems with a high dependence on training data and the need for appropriate selection of hyperparameters. To overcome this, we propose two approaches that use deep learning, a model by a custom architecture and a model by transfer learning. In each case, the impact of the depth of the network is analyzed in terms of precision (*P*), recall (*R*) and *F1* score. Additionally, the problem of generalization is addressed with images from eight different open access datasets. Finally, the models are compared in terms of evaluation metrics, and training and inference times. The model by transfer learning of VGG-16 achieves metrics about 10% higher than the model by a custom architecture, however, it requires approximately twice as much inference time as the latter.

## 1. Introduction

In recent years, the expansion of Internet services and the proliferation and strengthening of social platforms such as Facebook, Instagram and Reddit have had a significant impact on the amount of content circulating in digital media. According to the International Telecommunication Union (ITU), at the end of 2019, 53.6% of the world’s population uses the Internet, which means that approximately 4.1 billion people have access not only to this technology, but also to different tools available online [1]. Although in most cases the content shared is original or has only been manipulated for entertainment purposes, in other cases the manipulation may be intentional for disinformation purposes, with political and forensic repercussions, for example, using the fake content as digital evidence in a criminal investigation.

Image or video manipulation refers to any action that can be performed on a digital content using software editing tools (e.g., Adobe Photoshop, GIMP, PIXLR) or artificial intelligence. In particular, the copy-move technique copies a part of an image and then pastes it into the same image [2]. As editing tools advance, the quality of the fake images increases and they appear to the human eye as original images. In addition, post-processing manipulations, such as JPEG compression, brightness changes, or equalization, can reduce the traces left by manipulation and make it more difficult to detect [3].

The copy-move forgery detection (CMFD) includes hand-crafted and deep learning-based methods [2]. The former is mainly divided into block-based, keypoint-based and hybrid approach. The second uses custom architectures from scratch or fine-tuned models of pre-trained architectures such as VGG-16 [4]. Block-based approaches use different types of features extraction, for example, Fourier transform, DCT (Discrete Cosine Transform) [5,6] or Tetrolet transform [7]. One of their concerns is the reduction of performance when the copied object is rotated or resized, as the detection of counterfeiting is done through a matching process [8]. On the other hand, keypoint-based approaches such as SIFT (Scale Invariant Feature Transform) [9] and SURF (Speed-UP Robust Features) [10] are more robust to rotation and lighting variations but they have several challenges to overcome such as detecting counterfeits in regions of uniform intensity, natural duplicate objects detected as fake duplicate objects, and dependence on actual key points in the image [6]. A hybrid approach provided more stable results in terms of precision (*P*), recall (*R*) and *F1* score, but only for a single data set [11].

Recent approaches use Convolutional Neural Network (CNN) for feature extraction and classification [12,13]. For example, a custom CNN with nine convolutional layers and a fully connected (*FC*) layer was proposed in [14]. The architecture was trained separately with CASIA v1 and CASIA v2 datasets, obtaining an accuracy of 98.04% and 97.83%, respectively. A similar work used a custom model with six convolutional layers and three *FC* layers, with batch normalization in all the convolutional layers, and dropout in the *FC* layers (except the last one); using the CoMoFoD dataset, the internal validation of this model reached an accuracy of 95.97% [15]. A less deep custom architecture uses only two convolutional layers and two *FC* layers [16]. The authors trained and validated the model with one, two and three datasets obtaining *F1* scores of 90%, 94% and 95%, respectively. Although they address the problem of generalization, their mixed datasets are unbalanced, one with a ratio of 2:1 for fake and original images, and the other with a ratio of 2:3. Finally, a data-driven local descriptor with CNN obtained *F1* scores between 0.5 and 0.7 for the CoMoFoD dataset [17].

Complementary to custom designs with CNNs, a second group of CNN-based approaches use transfer learning (TL). In this case, pre-trained models are used even for feature extraction or by means of fine-tuning. For example, a pre-trained AlexNet model is followed by a feature comparison block and a post-processing stage which provides *F1* score of 93% for the GRIP dataset [18]. In other case, VGG-16 has been used as a feature extractor up to the last pooling layer [19]. Experiments were conducted with the MICC-F220 dataset reporting precision of 98%, recall of 89.5%, *F1* score of 92% and accuracy of 95%.

Although there are many proposals that have addressed the problem of CMFD, concerns and challenges remain as listed below:

- Most classification models using deep learning have been trained and validated with a unique dataset, limiting their use to other kind of tampered images. In other words, they have not addressed the problem of generalization;
- The CNN models trained with various datasets did not use class-balanced data, so they could be biased to a particular class;
- Most methods did not report image prediction times, so it is not possible to know if they are suitable in applications that require real time or massive data analysis;
- The deep learning-based works have used a single design strategy, either custom or by transfer learning, but, to our knowledge, no work has compared the two approaches for the same dataset.

According to the above, this research makes a contribution in the following aspects:

- Two models of CMFD based on deep learning are proposed, one corresponding to custom design and the other by transfer learning. Additionally, the dependence of the depth of the network on the performance of the classifier is evaluated.
- The generalization problem is addressed by training and validating the proposed models with data from eight public datasets. Also, external validation results are also compared when the architecture is trained on a single dataset.
- Finally, the inference time of the custom model is compared with that of the VGG-16 based model.

The rest of the paper is organized as follows—Section 2 presents the proposed architectures by custom design and transfer learning. Section 3 describes the experimental tests and the models selected for each design strategy. Section 4 presents the results for the generalization problem, as well as the comparison between the proposed models in terms of performance metrics and inference times. Section 5 discusses the results. Finally, Section 6 summarises the work.

## 2. Proposed Architectures for CMFD

We propose two approaches for CMFD. The first uses a custom design and the second uses transfer learning. Both design strategies are based on CNNs. In the following, we will explain each case.

### 2.1. Architectures by Custom Design

**Figure 1:**
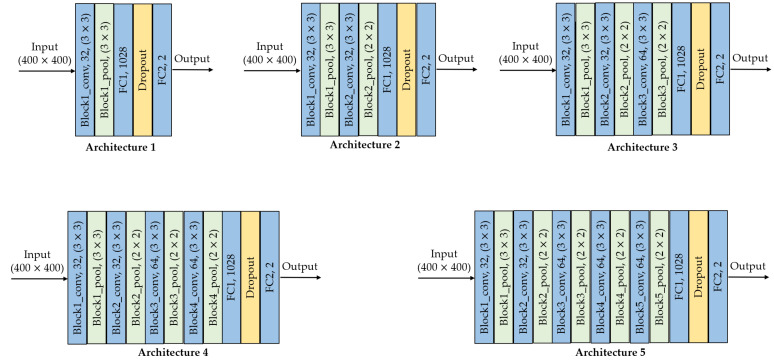
Architectures by custom design: *conv* is the convolutional layer, *pool* is pooling layer, *FC* is the fully-connected layer.

In this design strategy, we considered that the features that allow us to identify whether an image is original or has been manipulated with the copy-move technique are not found in very deep layers, since high-level features such as shape are not useful to solve this kind of problem. This is supported by previous studies, which state that in terms of network depth, the number of convolutional layers plus the number of *FC* layers, should not exceed 10 layers [14,15,16]. In our design, we propose five architectures with different depths, up to five convolutional layers (*conv*) with two *FC* layers. Figure 1 shows the proposed architectures, where each block specifies the type of layer (i.e., *conv*, pooling (*pool*) or *FC*), the number of filters and the size of the kernel. For example, *block1_conv*, 32, (3×3) is the first convolutional layer with 32 filters and kernel size of (3×3).

In all cases, the input image is resized to 400×400×3. The first convolutional layer has 32 filters of (3×3) size, with the Leaky_ReLU activation function using alpha = 0.09. In this layer, the padding is fixed as same, and the stride is 1 pixel. Its output is a feature map of 400×400×32, which has the same height *(H)* and width *(W)* of the input image, and the number of channels is equal to the number of filters. Next, the MaxPooling layer reduces the size of the input, that is, the feature map, in terms of *H* and *W*, but preserving the number of channels, according to Equation (1):

(1) $$H_{o}\times W_{o}=\left\lceil \left(H+2p-k+1\right)/s\right\rceil \times \left\lceil \left(W+2p-k+1\right)/s\right\rceil ,$$

where ⌈.⌉ is the ceiling function, *k* is the kernel size, *s* is the stride, *p* is the padding, $H_{o}$ is the height of the output, and $W_{o}$ is the weight of the output. With *H* = 400, *W* = 400, *p* = 0, *k* = 3 and *s* = 3, the output is $H_{o}\times W_{o}=\left\lceil \left(400-3+1\right)/3\right\rceil \times \left\lceil \left(400-3+1\right)/3\right\rceil =133\times 133$.

Hence, the difference between the architectures is the number of convolutional layers. The second architecture has two *conv*+*pool* layers; the third architecture has three *conv*+*pool* layers; and so on. All convolutional layers have padding equal to *same* so the *H* and *W* values of the input feature maps are preserved. Except for the first MaxPooling layer, these blocks work with k=2. The last two layers are fully-connected with 1028 and 2 units, respectively. Leaky_ReLU is used up to the penultimate layer, while softmax is used in the last layer.

Table 1 summarizes the hyperparameters of the five architectures by custom design. This table consists of four parts: the first part corresponds to the convolutional layers (from *block1_conv* to *block5_pool*); the second part corresponds to the *FC1* layer; the third part corresponds to the *FC2* layer; and the last part consolidates the five architectures.

Regarding activation function, Leaky_ReLU is chosen to avoid the dying ReLU problem. The alpha value (α) is fixed to 0.09 for convolutional layers, and 0.1 for the first *FC* layer. This activation function is calculated according to Equation (2).

(2) f(x)=max{αx,x},

where f(x) is the output of the activation function, and *x* is the input. Unlike the ReLU function, negative values are allowed at the output.

The second group of hyperparameters are related to the training stage. We have selected the following attributes: 80 epochs, categorical cross-entropy as loss function, SGD as the optimizer with a learning rate of 0.001 and a decay of 0.0001. Finally, with the purpose of reducing overfitting, we apply two strategies, dropout of 0.3 in the next-to-last layer, and image augmentation with horizontal and vertical flip.

### 2.2. Architectures by Transfer Learning (TL)

VGG-16 is one of the widely known models for image classification, which was trained with the sub-set of 1000 classes for the ImageNet Challenge [20]. The VGG architecture was proposed by the Visual Geometry Group of the University of Oxford and it is characterized by a stack of convolutional layers that precede a MaxPooling layer [21]. It has 3×3 and 1×1 filters, with a stride fixed to 1 pixel, and it includes 13 convolutional layers and 3 *FC* layers. Compared to the proposed architectures by custom design, VGG-16 differs in the depth of the network, in the number of filters in the convolutional layers, in the activation function, and also in the number of convolutional layers stacked before the pooling layer.

We have selected the pre-trained model VGG-16 for the following reasons:

- It is a sequential architecture as the proposed one, then we can compare the performance of the custom model with the model by transfer learning for the same type of architecture and analyze whether the features learned from a pre-trained model are useful for this type of classification problem;
- In recent work, some models by transfer learning with VGG-16 have been shown to be useful for identifying fake images from different types of manipulations such as copy-move [19] and colorization [12];
- Although VGG-16 is an architecture with a larger number of parameters and higher inference times than other architectures such as Inception or ResNet, it can be pruned for real-time applications without performance degradation [22].

Regarding transfer learning, there are several choices of using pre-trained models, one of them replaces part of the layers of the original architecture and preserves the others [13]. For instance, VGG-16 architecture can be preserved up to *block4_pool* layer and then add fully-connected layers with a different number of outputs. The new architecture will have pre-trained (frozen) parameters corresponding to those of the layers *block1_conv1* to *block4_pool*, and trainable parameters corresponding to the new *FC* layers.

**Figure 2:**
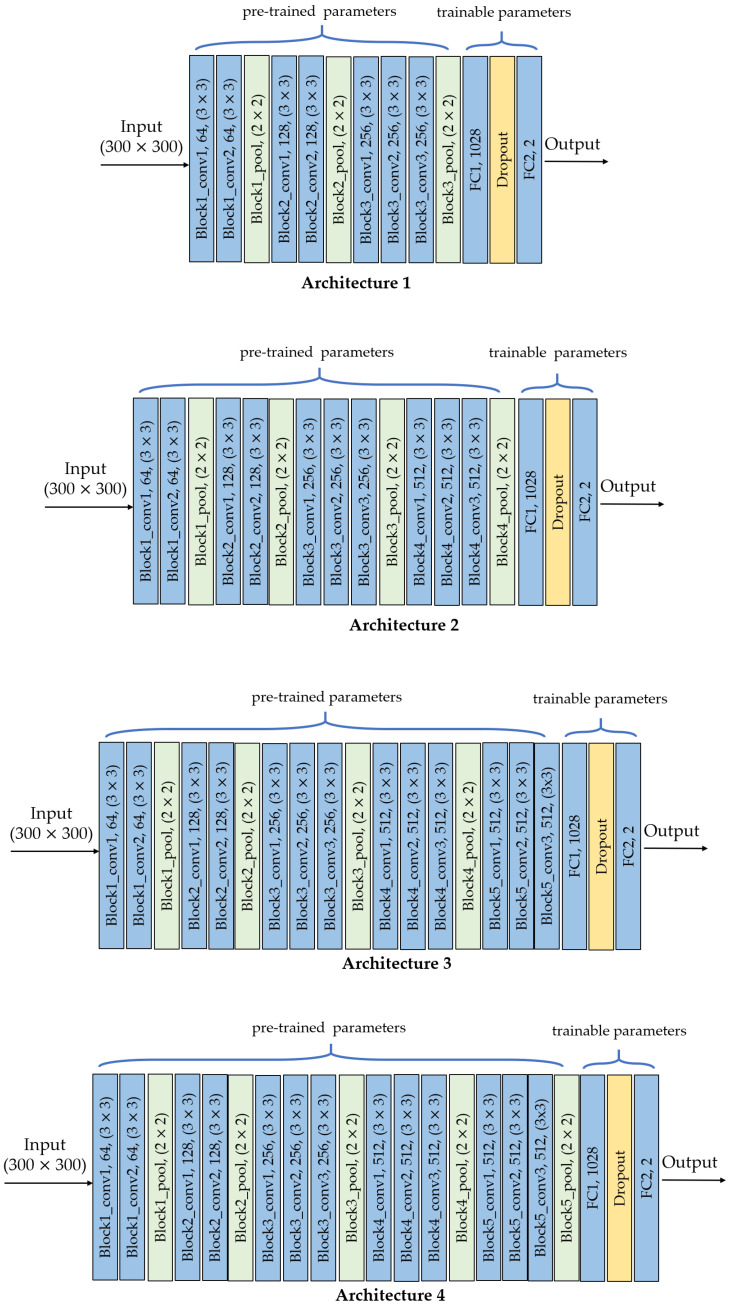
Architectures by TL: *conv* is convolutional layer, *pool* is pooling layer, *FC* is fully-connected layer.

In our case, we tested the performance of four different architectures from VGG-16, that is, different frozen points. Figure 2 shows the architectures with this design strategy and Table 2 shows their corresponding hyperparameters. The first part corresponds to the architecture that was transferred from VGG-16 with its pre-trained parameters. The second part corresponds to the *FC1* layer which depends on the frozen point. The third part is the *FC2* layer. The last part is the total number of parameters for each of the four architectures, which includes the pre-trained parameters and the trainable parameters (i.e., *FC1* + *FC2*). In all cases, the input image size is 300×300×3.

**Figure 3:**
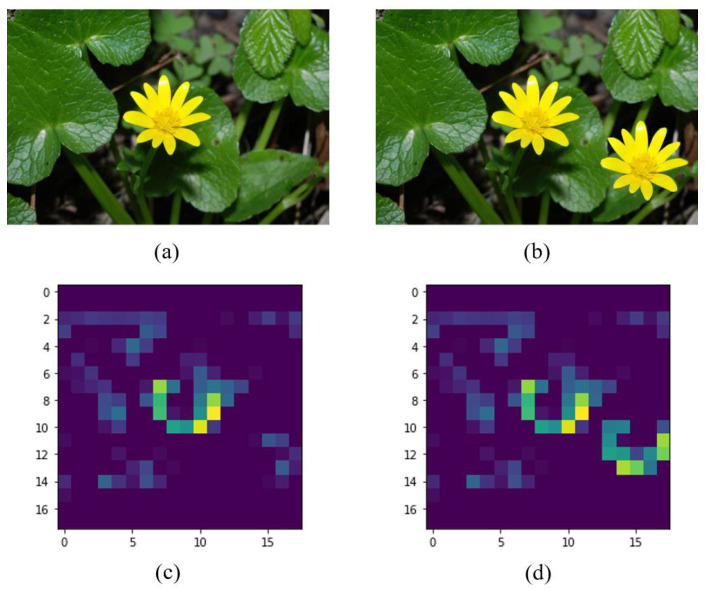
Example of feature maps for the *block4_pool* of the pre-trained VGG-16 model: (**a**) original image, (**b**) fake image with copy-move, (**c**) output of the filter 60 for the original image, (**d**) output of the filter 60 for the fake image. The source of (**a**,**b**) is [23] with permission of the authors.

On the other hand, to illustrate the features learned by the VGG-16 pre-trained model, we have selected the *block4_pool* layer to display one of its feature maps. Figure 3 shows the example for the filter 60. Similar patterns are detected in the feature maps for the two flowers, that is, a green and yellow semi-circle appears in the corresponding areas that have flowers. This type of pattern allows the classifier to make the decision on the originality of the image.

Finally, to train the transfer-learning based model, the same hyperparameters defined for the custom architecture are selected: 80 epochs, categorical cross-entropy as loss function, SGD as the optimizer with a learning rate of 0.001 and a decay of 0.0001. However, in this case, the dropout value is 0.45.

## 3. Experimental Tests

Bearing in mind that one of the purposes of this research was to address the problem of generalization, it is mandatory to consider several datasets for model training. Then, a unified dataset should have diversity in image size, format, color space and editing quality, that is, whether the manipulation is perceptible or not. This is done by unifying several datasets, some of which have been widely used in the literature, while others are new. Specifically, we have selected eight datasets, as follows: Coverage, CG-1050 v1, CG-1050 v2, MICC-F220, MICC-F2000, Copy-move Forgery Dataset (CMFD), CASIA v1 and CASIA v2. Table 3 shows the characteristics of each dataset.

The unified dataset contains images in JPEG, BMP and TIF formats, color and grayscale images, different sizes of the copied object and different orientation. They were split in training, validation, and testing (60%, 20% and 20%, respectively). Additionally, all images were converted to JPEG format, to train the model using images with a lossy compression format. Table 4 shows the number of fake and original images taken from each dataset.

It was necessary to add original images since some datasets only contained fake images. Table 5 shows the final distribution of the unified dataset, including 1308 original images obtained from a personal repository.

As shown in Table 5, the unified dataset is balanced by class, so that the models are not biased towards a particular class.

### 3.1. Comparison Metrics

To compare the performance of the classifier, we use three metrics that are suitable for balanced datasets, such as the unified dataset, corresponding to precision (*P*), recall (*R*) and *F1* score. Precision measures the ratio of images predicted as fake that belong to that class. Recall gives the ratio of fake images that are correctly classified. Finally, the *F1* score is the harmonic mean between precision and recall. These metrics are obtained using Equations (3)–(5):

(3) $$P=\frac{TP}{TP+FP},$$

(4) $$R=\frac{TP}{TP+FN},$$

(5) $$F1=2\times \frac{P\times R}{P+R},$$

where *TP* is the number of True Positives, *FP* is the number of False Positives and *FN* is the number of False Negatives. The class fake is the positive class while the class original is the negative class.

In addition, we use accuracy (*acc*) to compare our results with some of the state-of-the-art proposals. This metric is obtained with Equation (6):

(6) $$acc=\frac{TP+TN}{TP+FP+TN+FN},$$

The four metrics range between 0 and 1, their ideal value is 1 and the worst is 0.

### 3.2. Impact of the Depth for the Custom Architecture

**Figure 4:**
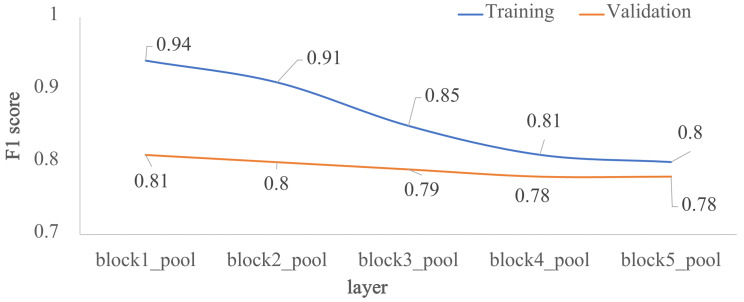
Impact of the depth for the custom architecture in terms of *F1* score: training vs validation. The *x*-axis corresponds to the last layer before the *FC1* layer, and the *y*-axis is the *F1* score.

**Figure 5:**
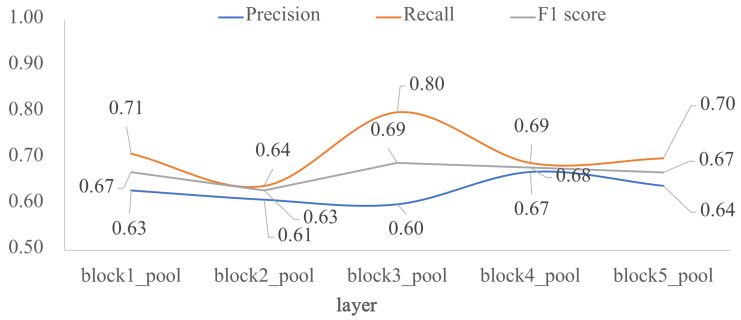
Impact of the depth for the custom architecture in terms of *P*, *R* and *F1* score: external test. The *x*-axis corresponds to the last layer before the *FC1* layer, and the *y*-axis are the evaluated metrics.

To assess how architecture depth affects model performance, we trained the five custom-designed architectures of Figure 1. As shown in Figure 4, model performance decreased as depth increased, the *F1* scores are very similar between layers *block4_pool* and *block5_pool* with a very low overfitting. The impact of the depth of the architecture was also analyzed in terms of external validation with the test dataset, that is, using images unknown to the trained model. Figure 5 shows the behavior of *F1* score, precision and recall by varying the number of convolutional layers. According to the results, *block2_pool* and *block4_pool* have the lowest variation in their metrics (*P*, *R*, *F1*), which means a better balance between precision and recall, however, the highest scores correspond to *block4_pool*.

Considering the above results, the proposed model by custom design was obtained from the architecture 4, that is, up to *block4_pool* with two *FC* layers.

### 3.3. Impact of the Depth for the Architecture by Transfer Learning

**Figure 6:**
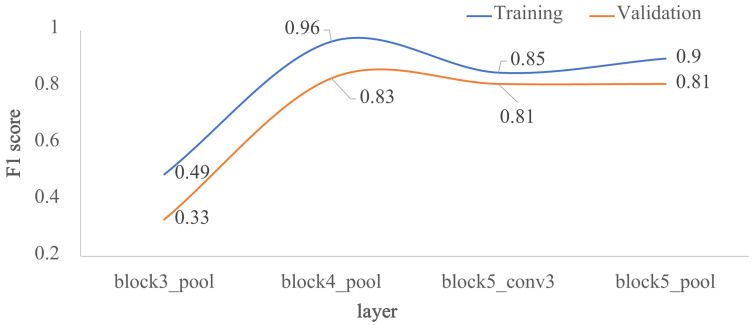
Impact of the depth for the architecture by transfer learning using VGG-16, in terms of *F1* score: training vs. validation. The *x*-axis corresponds to the selected last layer of VGG-16, and the *y*-axis is the *F1* score.

In the second approach, the four architectures in Figure 2 were evaluated, as it is shown in Figure 6. Each architecture corresponds to a specific freezing point of the pre-trained VGG-16 model: *block3_pool*, *block4_pool*, *block5_conv3* and *block5_pool*. The worst metrics were obtained for the most superficial freezing layer, where the metrics for training and validation did not exceed 0.5. Using deeper freezing points, the *F1* score in training exceeded 0.9 for *block4_pool* and *block5_pool* and 0.8 for *block5_conv3*. In validation, the *F1* score presented values of 0.83, 0.81 and 0.81 for *block4_pool*, *block5_conv3* and *block5_pool*, respectively. In summary, the *F1* score did not increase when increasing the network freezing point beyond the *block4_pool* layer.

**Figure 7:**
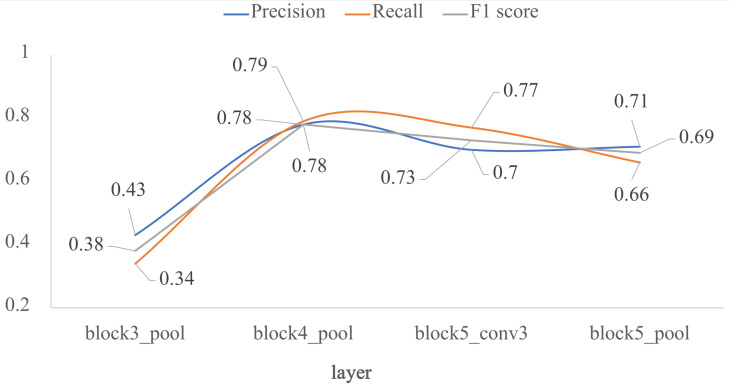
Impact of the depth for the architecture by transfer learning using VGG-16, in terms of *P*, *R* and *F1*: external test. The *x*-axis corresponds to the selected last layer of VGG-16, and the *y*-axis are the evaluated metrics.

Finally, the four models by transfer learning were validated with the test dataset. Figure 7 shows the results in terms of *F1* score. For the *block3_pool* layer, the best metric is precision, but for the *block5_conv3* layer the best metric is recall. The good balance between precision and recall is found in *block4_pool* layer in which *P*, *R*, and *F1* score are very close among them. This layer is a breaking point in the performance of the classifier. As the freezing point gets deeper, the performance metrics decrease, being lower than 0.71 for the *block5_pool* layer.

Therefore, the proposed model by transfer learning was obtained from the architecture 2, that is, up to *block4_pool* from VGG-16, plus two new *FC* layers.

## 4. Results

Considering that the TL-based model obtained better performance metrics than the custom-designed model (i.e., 0.78 vs 0.68 for *F1* score), the TL-based model is selected for the generalization tests in Section 4.1 and Section 4.2. In addition, both models are compared in terms of performance, training and inference times, in Section 4.3.

### 4.1. Training with a Single Dataset

**Figure 8:**
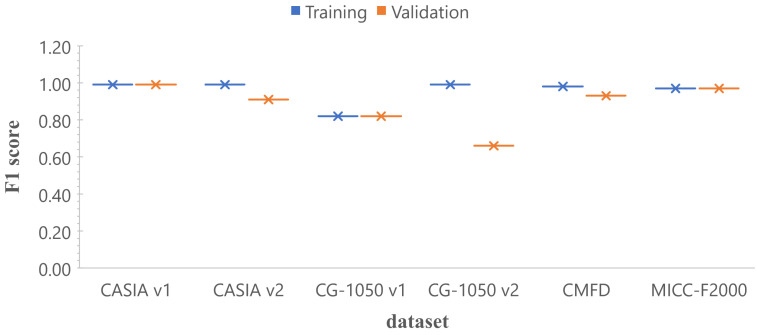
Comparison in terms of *F1* score with a single dataset (training vs. validation). The *x*-axis corresponds to the model trained with the specific dataset, and the *y*-axis is the *F1* score.

In this section we trained the architecture 2 of the TL-based strategy with six different datasets, (i.e., CASIA v1, CASIA v2, CG-1050 v1, CG-1050 v2, Copy-move forgery dataset (CMFD) and MICC-F2000), obtaining six different models. Figure 8 shows the results in terms of *F1* score for training and validation. It should be noted that, for the datasets of CASIA v1, MICC-F2000 and CG-1050 v1, *F1* scores are very close to each other for training and validation, and close to 1; while for CASIA v2 and CMFD, the values are higher than 0.9. Only, for the CG-1050 v2 dataset, the validation value is distant from the training one. In summary, the same architecture is able to obtain high scores for different datasets when the model is validated with data similar to those used in the training stage.

The next step is to compare the results with those reported in the literature with a single dataset. Table 6 shows either the *acc* or the *F1* score for some works, divided in three groups: CASIA (v1, v2), CMFD and MICC (F2000, F220, F600). For the copy-move forgery dataset (CMDF) group, the results were very similar between them. For the CASIA group, similar results are found for custom CNN and by transfer learning models. For the MICC group, deep learning-based methods outperform hand-crafted based methods. However, it is not clear whether the classifiers are biased to one class, as few papers report all four metrics: *acc*, *P*, *R* and *F1*. For instance, in [29] the authors reported a precision of 89.0% and recall of 100%, which implies that these metrics are not balanced, and specifically, the classifier is slightly biased to the positive class.

Thus, our proposed TL-based model exceeds most of the results reported by other works, in two of the three groups of this comparison. It should be noted that transfer learning with VGG-16 had already been used for the same classification task (i.e., ref [19]) up to the *block5_pool* layer, but its results in terms of *F1* score are lower than those of our work. This is because after the *block4_pool* layer the performance decreases, as is reported in Figure 7.

### 4.2. Adressing the Problem of Generalization

**Figure 9:**
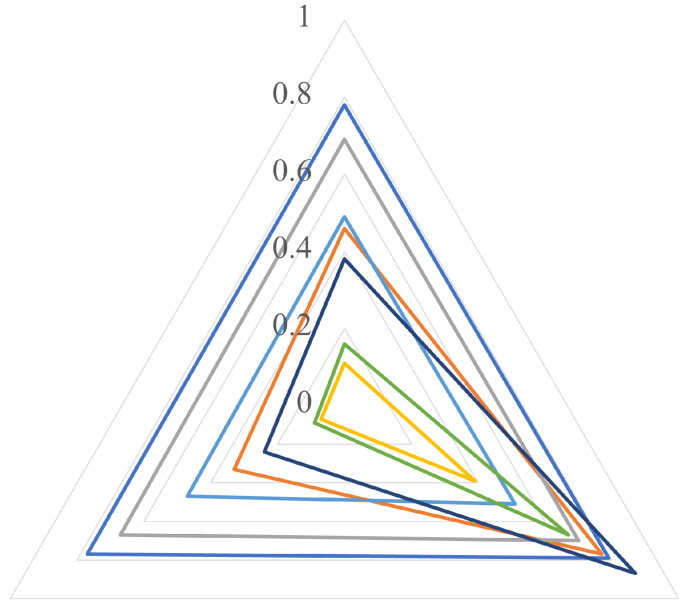
Ability of generalization: single dataset vs. unified dataset (UD). The triangle represents *P* (up), *R* (down, right) and *F1* score (down, left).

We evaluate the generalization capacity when the architecture by transfer learning is trained with a single dataset versus several datasets. We test the six models trained with a single dataset (Section 4.1) against the unified dataset, and we compare their results with the selected model of Section 3.3. Figure 9 shows a radar plot in which each vertex of the triangle corresponds to *P* (up), *R* (down, right) and *F1* score (down, left). The best model is the one whose curve is the most external, without biasing any of the metrics.

As shown in Figure 9, the outermost curve corresponds to the model trained with the unified dataset, in which there is an adequate balance between *P* and *R*, and therefore the three values (*P*, *R* and *F1* score) are very similar between them. The second place is occupied by the model trained with CASIA v2 in which again the metrics are balanced but are lower than in the first case. One of the worst curves was found with the model trained with CMFD dataset, in which *R* is high but *P* is low. This means that few fake images are labelled as original, but many original images are labelled as fake.

According to the above, not only the results for individual datasets are important to know the quality of the model, but also the generalization results. An architecture trained and evaluated on a single dataset can achieve high *F1* score values, but it is no guarantee of high performance for new images.

### 4.3. Custom Model vs. Vgg-16 Based Model

In this last test section, we compare the two proposed models in terms of performance (Table 7), number of parameters (Table 8) and training and inference times (Table 9).

In terms of performance, the model by transfer learning shows higher scores in all four statistics. However, in both models, there is a high trade-off between accuracy and recall, and therefore, the amount of misclassifications is similar for the two classes (original and fake).

In the second comparison, all parameters are trainable in the custom architectures, while only the *FC* layer parameters are trainable in the architectures by transfer learning. In the first case, architecture 4 from Table 1 was selected, while in the second case, architecture 2 from Table 2 was selected. As shown in Table 8, the total number of parameters of the second approach is much higher than the former.

On the other hand, in terms of training and inference times (Table 9), the model by custom architecture uses about 65% of that of the transfer-learning-based.

In summary, the model by the custom architecture has a lower number of parameters than the model by transfer learning, with less inference time, but with a lower success rate in the classification.

## 5. Discussion

This research addressed the generalization problem, where models trained with a single dataset can show very high results with similar data, but their performance decreases significantly with data dissimilar to that of the training stage. In most papers, copy forgery detection models have been trained and validated (internally) with a single dataset or with datasets from the same “family”, for example, CASIA (v1 and v2) or MICC (2000, F220 F600), but not with data from multiple and highly diverse datasets.

Fitting the model for single datasets is not a very difficult task, as presented in Figure 8, where the same architecture was used to train six single models with high results in five of the six cases. However, in a real scenario, the trained models must classify images dissimilar to those used in the training stage, and then, models trained with a single data set do not perform adequately, as was shown in Figure 9. For instance, the model trained with the CMFD dataset obtained *F1* scores close to 1 when evaluated with images from the same dataset, but its performance decreased when tested with images from other datasets showing high recall and very low precision.

For this reason, to approach a real scenario, we used a unified dataset from different individual datasets, some of which have already been used in other works and others are new, with a high diversity as summarized in Table 3. Obtaining a trained model with high precision and recall, and a proper balance between these two metrics was not an easy task, so it was necessary to evaluate several hyperparameters. After several adjustments of the hyperparameters (both related to the architecture as well as the training stage) we obtained two promising models, which are presented in this paper.

It is worth noting that the task of copy-move forgery detection is not yet solved, because every day in social networks new high quality manipulated images are found, which could be classified not only by an algorithm but by human beings as originals.

## 6. Conclusions

In this paper we proposed two deep learning-based models, a custom model and a TL-based model, to evaluate its effectiveness in the CMFD task. Additionally, we address the generalization problem when the architecture is trained only with one dataset but tested with several datasets versus the approach trained with a large dataset. Finally, we evaluate not only the performance of the proposed models but their training and inference times. According to our results, the custom architecture with few convolutional layers have greater generalization problems than those with more layers; however, in the VGG-16 pre-trained model it was found that when using a freezing point beyond the *block4_pool* layer the classifier results get worse. Additionally, it was found that models trained with a single dataset tend to classify images into a single class (original or fake), such that *P* and *R* metrics are not balanced (one high and one low). The best balance between these metrics was obtained when the weights of the pre-trained VGG-16 model were frozen in the *block4_pool* layer and the unified dataset was used. Besides, the improved performance in classifying original and fake images in the VGG-16 based model has a direct relationship with the inference time, which is almost double that of the custom model.

Future work may consider extending the training dataset, assessing the impact of other hyperparameters on classifier performance and a hybrid approach mixing an image pre-processing stage using domain transformation (e.g., DFT, DCT, and DWT) with feature extraction based on deep learning.

## Figures and Tables

**Table 1:** Hyperparameters of the proposed architectures by custom design: *k* is the kernel size, *s* is stride, *p* is padding.

Layer	No. of Filters or Neurons	*k*	*s*	*p*	Output Shape	Trainable Parameters
Input	–	–	–	–	(400, 400, 3)	0
*block1_conv*	32	3	1	same	(400, 400, 32)	896
*block1_pool*	32	3	3	0	(133, 133, 32)	0
*block2_conv*	32	3	1	same	(133, 133, 32)	9248
*block2_pool*	32	2	2	0	(66, 66, 32)	0
*block3_conv*	64	3	1	same	(66, 66, 64)	18,496
*block3_pool*	64	2	2	0	(33, 33, 64)	0
*block4_conv*	64	3	1	same	(33, 33, 64)	36,928
*block4_pool*	64	2	2	0	(16, 16, 64)	0
*block5_conv*	64	3	1	same	(16, 16, 64)	36,928
*block5_pool*	64	2	2	0	(8, 8, 64)	0
*FC1* (architecture 1)	1028	–	–	–	–	581,898,372
*FC1* (architecture 2)	1028	–	–	–	–	143,296,004
*FC1* (architecture 3)	1028	–	–	–	–	71,648,516
*FC1* (architecture 4)	1028	–	–	–	–	16,843,780
*FC1* (architecture 5)	1028	–	–	–	–	4,211,716
*FC2*	2	–	–	–	–	2058
Architecture 1	–	–	–	–	–	581,901,326
Architecture 2	–	–	–	–	–	143,308,206
Architecture 3	–	–	–	–	–	71,679,214
Architecture 4	–	–	–	–	–	16,911,406
Architecture 5	–	–	–	–	–	4,316,270

**Table 2:** Hyperparameters of the architectures by transfer learning: *k* is the kernel size, *s* is stride, *p* is padding.

Layer	No. of Filters or Neurons	*k*	*s*	*p*	Output Shape	Trainable Parameters
Input	–	–	–	–	(400, 400, 3)	0
*block1_conv1*	64	3	1	same	(300, 300, 64)	1792
*block1_conv2*	64	3	1	same	(300, 300, 64)	36,928
*block1_pool*	64	2	2	0	(150, 150, 64)	0
*block2_conv1*	128	3	1	same	(150, 150, 128)	73,856
*block2_conv2*	128	3	1	same	(150, 150, 128)	147,584
*block2_pool*	128	2	2	0	(75, 75, 128)	0
*block3_conv1*	256	3	1	same	(75, 75, 256)	295,168
*block3_conv2*	256	3	1	same	(75, 75, 256)	590,080
*block3_conv3*	256	3	1	same	(75, 75, 256)	590,080
*block3_pool*	256	2	2	0	(37, 37, 256)	0
*block4_conv1*	512	3	1	same	(37, 37, 512)	1,180,160
*block4_conv2*	512	3	1	same	(37, 37, 512)	2,359,808
*block4_conv3*	512	3	1	same	(37, 37, 512)	2,359,808
*block4_pool*	512	2	2	0	(18, 18, 512)	0
*block5_conv1*	512	3	1	same	(18, 18, 512)	2,359,808
*block5_conv2*	512	3	1	same	(18, 18, 512)	2,359,808
*block5_conv3*	512	3	1	same	(18, 18, 512)	2,359,808
*block5_pool*	512	2	2	0	(9, 9, 512)	0
*FC1* (architecture 1)	1028	–	–	–	–	360,278,020
*FC1* (architecture 2)	1028	–	–	–	–	170,533,892
*FC1* (architecture 3)	1028	–	–	–	–	170,533,892
*FC1* (architecture 4)	1028	–	–	–	–	42,634,244
*FC2*	2	–	–	–	–	2058
Architecture 1	–	–	–	–	–	362,015,566
Architecture 2	–	–	–	–	–	178,171,214
Architecture 3	–	–	–	–	–	185,250,638
Architecture 4	–	–	–	–	–	57,348,932

**Table 3:** Characteristics of the selected datasets.

Dataset	Grayscale	Color	TIFF	JPEG	BMP	Original	Fake
COVERAGE [24]		x	x			x	x
CG-1050 v1 [25]	x	x		x		x	x
CG-1050 v2 [26]	x	x		x		x	x
MICC-F220 [27]		x		x			x
MICC-F2000 [27]		x		x		x	x
CMFD dataset [23]		x			x	x	x
CASIA v1 [28]		x		x			x
CASIA v2 [28]		x		x			x

**Table 4:** Unified dataset: number of images taken from single datasets.

Dataset	Fake	Original
COVERAGE	100	99
CG-1050-V1	331	100
CG-1050-V2	328	1044
MICC-F220	29	0
MICC-F2000	700	346
CMFD	383	50
CASIA V1	370	0
CASIA V2	706	0

**Table 5:** Distribution of the unified dataset: training, validation, and test.

Dataset	Training	Validation	Test
Fake	1765	590	592
Original	1766	590	591

**Table 6:** Comparison between state-of-the-art approaches in terms of accuracy (acc) and *F1* score for the datasets: CASIA (v1, v2), CMFD and MICC (F2000, F220, F600). The higher the better.

Method/Year/Reference	CASIA	CMFD	MICC	*acc*	*F1*
CNN (9 *conv* layers)/2016/[14]	x			98.0%	
Our–VGG-16 based/2020	x			98.0%	99.0%
Keypoint clustering/2020/[30]		x			93.8%
Our–VGG-16 based/2020		x		94.0%	93.0%
VGG-16 based (*block5_pool*)/2019/[19]			x	95.0%	92.0%
CNN (6 *conv* layers)/2020/[31]			x	99.5%	
CNN (3 *conv* layers, dual branch)/2021/[29]			x	96.0%	94.0%
Hand-crafted (feature point)/2016/[32]			x		74.0%
Hand-crafted (hybrid feature extraction)/2020/[11]			x		93.0%
Our–VGG-16 based/2020			x	97.0%	97.0%

**Table 7:** Comparison of the two proposed models in terms of *acc*, *P*, *R* and *F1* score.

Model	*acc*	*P*	*R*	*F1*
Model by custom design	0.68	0.67	0.69	0.68
Model by transfer learning	0.78	0.78	0.79	0.78

**Table 8:** Comparison of the two proposed models in terms of number of parameters.

Model	Parameters	Trainable	Non-Trainable
Model by custom design	16,911,406	16,91,406	0
Model by transfer learning	178,171,214	170,535,950	7,635,264

**Table 9:** Comparison of the two proposed models in terms of training and validation times, *h* is hours, *sec* is seconds.

Model	Training Time (h)	Inference Time by Image (sec)
Model by custom design	1.8	0.0354
Model by transfer learning	2.8	0.0532

## Data Availability

The data CG-1050 used in this study are openly available in [25,26].

## Abbreviations

The following abbreviations are used in this manuscript:

*acc*	Accuracy
CNN	Convolutional Neural Network
CMFD	Copy-Move Forgery Detection
*conv*	Convolutional layer
DCT	Discrete Cosine Transform
DFT	Discrete Fourier Transform
DWT	Discrete Wavelet Transform
*FC*	Fully-Connected
*P*	Precison
*pool*	Pooling layer
*R*	Recall
SIFT	Scale Invariant Feature Transform
SURF	Speed-Up Robust Features
